# Radiation Dose-Response for Risk of Myocardial Infarction in Breast Cancer Survivors

**DOI:** 10.1016/j.ijrobp.2018.10.025

**Published:** 2019-03-01

**Authors:** Judy N. Jacobse, Frances K. Duane, Naomi B. Boekel, Michael Schaapveld, Michael Hauptmann, Maartje J. Hooning, Caroline M. Seynaeve, Margreet H.A. Baaijens, Jourik A. Gietema, Sarah C. Darby, Flora E. van Leeuwen, Berthe M.P. Aleman, Carolyn W. Taylor

**Affiliations:** ∗ Department of Psychosocial Research and Epidemiology, Netherlands Cancer Institute, Amsterdam, The Netherlands; † Medical Research Council Population Health Research Unit, Nuffield Department of Population Health, University of Oxford, Oxford, United Kingdom; ‡ Department of Biostatistics, Netherlands Cancer Institute, Amsterdam, The Netherlands; § Department of Medical Oncology, Erasmus MC, Cancer Institute, Rotterdam, The Netherlands; ‖ Department of Radiation Oncology, Erasmus MC, Cancer Institute, Rotterdam, The Netherlands; ¶ Department of Medical Oncology, University Medical Center Groningen, Groningen, The Netherlands; ∗∗ Clinical Trial Service Unit, Nuffield Department of Population Health, University of Oxford, Oxford, United Kingdom; †† Department of Radiation Oncology, Netherlands Cancer Institute, Amsterdam, The Netherlands

## Abstract

**Purpose:**

Previous reports suggest that radiation therapy for breast cancer (BC) can cause ischemic heart disease, with the radiation-related risk increasing linearly with mean whole heart dose (MWHD). This study aimed to validate these findings in younger BC patients and to investigate additional risk factors for radiation-related myocardial infarction (MI).

**Methods and Materials:**

A nested case-control study was conducted within a cohort of BC survivors treated during 1970 to 2009. Cases were 183 patients with MI as their first heart disease after BC. One control per case was selected and matched on age and BC diagnosis date. Information on treatment and cardiovascular risk factors was abstracted from medical and radiation charts. Cardiac doses were estimated for each woman by reconstructing her regimen using modern 3-dimensional computed tomography planning on a typical patient computed tomography scan.

**Results:**

Median age at BC of cases and controls was 50.2 years (interquartile range, 45.7-54.7). Median time to MI was 13.6 years (interquartile range, 9.9-18.1). Median MWHD was 8.9 Gy (range, 0.3-35.2 Gy). MI rate increased linearly with increasing MWHD (excess rate ratio [ERR] per Gy, 6.4%; 95% confidence interval, 1.3%-16.0%). Patients receiving ≥20 Gy MWHD had a 3.4-fold (95% confidence interval, 1.5-7.6) higher MI rate than unirradiated patients. ERRs were higher for younger women, with borderline significance (ERR_<45years_, 24.2%/Gy; ERR_≥50years_, 2.5%/Gy; *P*_interaction_ = .054). Whole heart dose-volume parameters did not modify the dose-response relationship significantly.

**Conclusions:**

MI rate after radiation for BC increases linearly with MWHD. Reductions in MWHD are expected to contribute to better cardiovascular health of BC survivors.

**Summary**

Previous reports suggest that radiation therapy for breast cancer can cause ischemic heart disease, with radiation-related risk increasing linearly with mean whole heart dose. This study aimed to validate these findings and assesses additional risk factors for radiation-related myocardial infarction in a case-control study nested within a cohort of BC survivors treated ≤70 years of age during 1970-2009. The study confirms a linear relationship between mean whole heart dose and myocardial infarction risk after radiation for breast cancer.

## Introduction

Survival in women with breast cancer (BC) has improved substantially in recent decades as a result of more effective treatments and earlier diagnoses.^1^, ^2^, ^3^, ^4^, ^5^ However, radiation therapy, anthracycline-based chemotherapy, and targeted therapies can cause heart disease.^6^, ^7^, ^8^ In the case of radiation therapy, cardiac exposure is associated with an increased risk of ischemic heart disease (IHD).^9^, ^10^, ^11^ This may occur through injury to the small vessels in the heart muscle (microvascular damage) or through atherosclerosis of the larger blood vessels (macrovascular damage).^12^, ^13^

The risk of IHD appears to increase linearly with increasing mean whole heart radiation dose (MWHD).^14^, ^15^ In a large case-control study of women irradiated for BC, the rate of major coronary events increased by 7.4%/Gy MWHD.^14^ The increase started within 5 years of exposure, sooner than previously anticipated.^16^, ^17^ It was also found that the percentage increase in IHD per Gy was similar in women with and without cardiovascular risk factors, suggesting that the absolute radiation-related increase is higher in those with preexisting cardiovascular risk factors. These associations require further study in younger women (ie, <50 years) diagnosed with BC.

Substantial progress has been made in reducing cardiac radiation doses in BC radiotherapy.^18^, ^19^, ^20^ Nevertheless, some recent regimens used to irradiate the left internal mammary nodes still may expose the heart to ≈8 Gy.^21^ Quantification of the effect of radiation on the heart may help to predict IHD risk for women being considered for BC radiation therapy today. It may also help to identify BC survivors who may benefit from cardiac surveillance.

This study aimed to examine the radiation dose-response relationship for myocardial infarction (MI) in a population of younger BC patients (median age 50 years at BC diagnosis). It also investigates the effects of different whole heart dose-volume radiation measures and cardiovascular risk factors on the dose-response relationship.

## Methods

### Cohort population

A nested case-control study of MI after BC was conducted within a cohort of long-term BC survivors treated in the Netherlands between 1970 and 2009. The cohort was identified through hospital-based registries at the Netherlands Cancer Institute, Amsterdam and Erasmus MC-Cancer Institute, Rotterdam, the Netherlands. Female patients were included in the cohort if they had histologically confirmed, stage I to III invasive BC or ductal carcinoma in situ and were diagnosed before the age of 71 years. Details of the data collection procedures have been published previously^10^, ^22^ and are outlined in Methods E1 (available online at https://doi.org/10.1016/j.ijrobp.2018.10.025). In brief, for patients in the cohort, information on BC recurrence, distant metastases, second cancer incidence, cardiovascular disease incidence, cardiovascular risk factors, and causes of death was obtained through registries, medical files, general practitioners, and cardiologists.

### Case-control study

MI diagnoses in the cohort were ascertained from medical files and through questionnaires sent to the general practitioner. In the Netherlands, all residents are expected to have a general practitioner. Medical correspondence from attending physicians is sent to the general practitioner. Such records are preserved by the general practitioner throughout a patient's life and for at least 15 years after a patient's death. When the treating cardiologist was known and event information obtained from the general practitioner/medical file was unclear, an additional questionnaire was sent to the cardiologist to confirm the MI diagnosis. Patients with MI after BC were eligible as cases if they met the following criteria: (1) BC was the first invasive malignancy (ignoring nonmelanoma skin cancer); (2) they were free from BC recurrence, distant metastases, second cancer, heart failure, and valvular heart disease when diagnosed with MI. Heart failure and valvular heart disease were addressed in separate case-control studies. A rhythm disorder or angina pectoris before MI was not an exclusion criterion. In total 183 patients with a diagnosis of MI after BC were selected as cases.

For each MI case, one control was selected from the cohort, matched on age at BC diagnosis (±5 years) and date of BC diagnosis (±5 years). Only patients for whom we could collect information on cardiovascular disease were considered as potential controls. Controls had to be alive and free from BC recurrence, distant metastases, second cancer, MI, heart failure, and valvular heart disease until the cutoff date, which was defined as the date of BC diagnosis plus a time interval equal to the time between BC diagnosis and MI diagnosis for the paired case. Cases were permitted to be controls up to the date when they developed MI, and controls were selected with replacement. In total, 183 controls (178 unique individuals) were matched to the cases. Four controls later became a case.

### Data collection

Data at BC diagnosis regarding medical history (including prior cardiovascular diseases, diabetes, and hypertension), smoking history, and body mass index (BMI) were abstracted from medical files. Hypertension was coded as present if a patient was on medication for hypertension or if a diagnosis of hypertension was given in the medical record. Information on cardiovascular risk factors diagnosed after BC diagnosis was also collected. Treatment information, including surgery, chemotherapy regimen, and endocrine therapy, was abstracted. Copies of radiation charts for individual patients were obtained for patients who received radiation therapy. Information on target definition, field borders, total dose, dose per fraction, beam energy, and the use of shielding, wedges, and bolus was obtained from the charts.

### Radiation therapy dosimetry

A ‘typical computed tomography (CT) scan’ was used to estimate cardiac doses retrospectively for each woman in the study (Methods E2; available online at https://doi.org/10.1016/j.ijrobp.2018.10.025). Radiation therapy charts were available for 314 of the 316 individual irradiated women in the study. Fifty-two regimens were identified and reconstructed on the typical CT scan (Table E1; available online at https://doi.org/10.1016/j.ijrobp.2018.10.025). Dose distributions were generated for cobalt, electron, and megavoltage beams using modern 3-dimensional CT treatment planning (Varian EclipseTM Treatment Planning System Version 10.0.39, Varian Medical Systems, Palo Alto, CA). Dose distributions from orthovoltage fields were generated using manual planning. Typical MWHD, mean left ventricle dose, and the percentage volume of the heart receiving ≥5, 10, 15, 20, 25, 30, 35, and 40 Gy (V_5_-V_40_) were estimated using dose-volume histograms. Doses were estimated for each individual woman using the total dose (100%) received as recorded in individual radiation therapy charts and the dose-volume histogram of the regimen received.

### Statistical analysis

Rate ratios (RRs) for MI for different levels of each factor were calculated using logistic regression conditioning on strata. Strata were defined by the matching variables age at BC diagnosis (10-year categories), year of BC diagnosis (10-year categories), and follow-up duration (5-year categories). One individual, who appeared as a control twice within the same stratum, was included only once in the analysis.

Confidence intervals (CIs) were estimated using the Wald method for factors with 2 levels. For factors with more than 2 levels, Wald method CIs were used to derive CIs for each category, including the reference category, from the amount of information in that category.^23^ Multivariable regression was used to assess and control for potential confounders. A factor was considered a potential confounder if the RR after adjustment for the factor changed by 10% or more.

The excess rate ratio (ERR) of MI associated with a one Gy increase in radiation exposure (ie, the proportional increase in MI rate per unit increase in dose) was estimated using a linear odds model conditional on strata defined by the matching variables. The model for the MI rate was Κ_m_(1 + βd) where d is MWHD for each woman, Κ_m_ is the MI rate at zero MWHD in the m^th^ stratum, and β is the ERR. Nonlinearity was tested by adding an exponential term: Κ_m_(1 + βd*exp[δd]). The significance of the dose-response relationship was evaluated using the likelihood ratio test. Interactions were evaluated by including interaction terms as categorical or continuous variables.

Approximate cumulative MI risks and 95% CIs were estimated from the treatment-specific RRs together with the cumulative MI risk for the entire cohort. Death from any cause other than MI or sudden cardiac death was treated as a competing risk. Overall survival rates after a diagnosis of MI were estimated using the Kaplan-Meier method with cases censored at either last known follow-up date or date of death. Analyses were performed using Stata Statistical Software version 13.0 (StataCorp LP, College Station, TX) and Epicure version 1.8 (Hirosoft International, Eureka, CA).

## Results

### Patient characteristics

The median age of cases at BC diagnosis was 50.2 years (interquartile range [IQR], 45.8-54.7; Table 1). Median time between BC diagnosis and MI diagnosis was 13.6 years (IQR, 10.3-18.2). Left-sided BC was more common than right-sided BC in cases and controls (53.6% and 55.5% left-sided vs 46.4% and 44.5% right-sided, respectively) and 57.9% of cases and 58.8% of controls had node-negative disease at BC diagnosis; these differences were not statistically significant (Table 1). Five year and 15-year cumulative survival among cases were 85% and 56%, respectively (Fig. E1; available online at https://doi.org/10.1016/j.ijrobp.2018.10.025). Twenty-three patients (13%) died on the day of MI diagnosis.

**Table 1:** Characteristics of myocardial infarction cases and matched controls

Total	Cases		Controls		*P*‡
	183 (N)	100 (%)∗	182† (N)	100 (%)∗	
Age at breast cancer diagnosis§					
Median age (IQR) in years	50.2	45.8-54.7	50.2	45.5-54.6	
<40 (22-39) y	9	4.9	10	5.5	
40-49 y	78	42.6	78	42.9	
50-59 y	77	42.1	75	41.2	
≥60 (60-70) y	19	10.4	19	10.4	
Year of breast cancer diagnosis§					
1970-1979	41	22.4	41	22.5	
1980-1989	95	51.9	92	55.6	
1990-1999	29	15.9	34	18.7	
2000-2009	18	9.8	15	8.2	
Time to MI/cutoff date§,¶					
Median time (IQR) in years	13.6	10.3-18.2	13.7	10.3-18.2	
<5 (1-4) y	13	7.1	13	7.1	
5-9 y	31	16.9	31	17.0	
10-14 y	60	32.8	59	32.4	
15-19 y	48	26.2	48	26.4	
≥20 (20-29) y	31	16.9	31	17.0	
Age at MI diagnosis/cutoff date¶					
Median age (IQR) in years	64.2	58.5-70.2	63.9	58.5-70.2	
<50 (40-49) y	7	3.8	8	4.4	
50-59 y	51	27.9	50	27.5	
60-69 y	74	40.4	74	40.7	
70-79 y	45	24.6	44	24.2	
≥80 (83-89) y	6	3.3	6	3.3	
Laterality of breast cancer					
Right	85	46.4	81	44.5	
Left	98	53.6	101	55.5	.72
Nodal status					
Negative	106	57.9	107	58.8	
Positive	75	41.0	74	40.7	
Unknown	2	1.1	1	0.5	.91
Tumor size∗					
<2 cm	58	31.7	76	41.8	
2-5 cm	88	48.1	72	39.6	
≥5 cm	6	3.3	10	5.5	
Unknown	31	16.9	24	13.2	.09

*Abbreviations:* BC = breast cancer; IQR = interquartile range; MI = myocardial infarction.

∗ Percentages may not total 100 because of rounding.

† Cases and controls were selected with replacement. When an individual appeared as a control twice within the same stratum, it was included only once in the analysis (n = 1).

‡ *P* value for difference between tumor characteristics of cases and controls, calculated within strata (defined by matching variables) and excluding the unknown category.

§ Matching variables.

¶ Derived from matching factors. Cutoff date for controls was defined as the date of BC diagnosis plus a time interval equal to the time between BC diagnosis and MI diagnosis for the paired case.

### Treatment-related risk factors

**Fig. 1:**
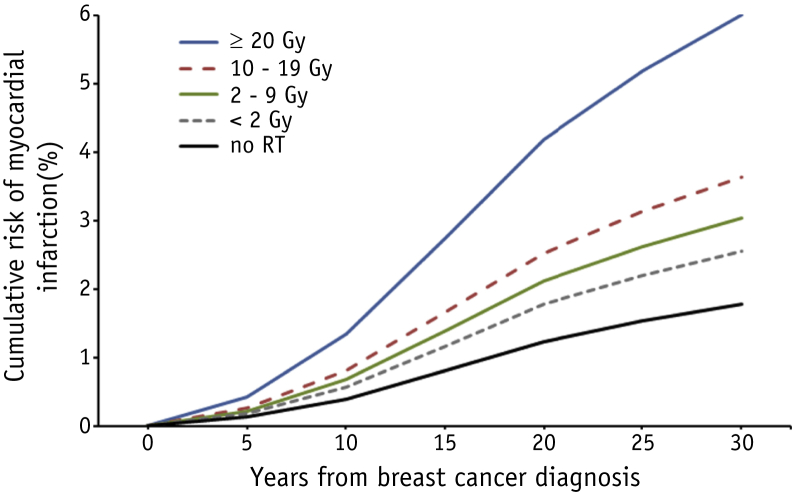
Modeled cumulative myocardial infarction risk for cases by categories of mean whole heart dose. Cumulative risks of myocardial infarction (MI) as a first cardiac event among women diagnosed with breast cancer (BC) when aged ≤70 years (median age at BC diagnosis, 50.2 years (interquartile ratio [IQR], 45.8-54.7) and median age at MI diagnosis, 64.1 years (IQR, 58.5-70.2)) by time since initial BC treatment for categories of mean whole heart dose. Cumulative risks were calculated from the MI rate ratios for different dose categories (Table 2) and the cumulative MI risk for the entire BC cohort in which this study is embedded, with death as a competing event. *Abbreviation:* RT = radiation therapy.

Surgery, endocrine therapy, and chemotherapy (with or without anthracyclines) were not associated with a significantly increased MI rate (RR mastectomy vs breast-conserving surgery, 1.04 [95% CI, 0.58-1.86]; RR hormone therapy vs not, 0.71 [95% CI, 0.29-1.75]; RR non-anthracycline chemotherapy vs no chemotherapy, 0.59 [95% CI, 0.33-1.07], and RR anthracyclines vs no chemotherapy, 1.04 [95% CI, 0.41-2.63]; Table 2). Internal mammary chain (IMC) irradiation was associated with a 2.45-fold increased MI rate (95% CI, 1.97-3.05). Median MWHD was 8.9 Gy (IQR, 4.8-15.0) for cases and 8.5 Gy (IQR, 4.3-12.2) for controls (Table 2). Heart dose estimates were highest for patients who received radiation to the IMC only or IMC combined with breast or chest wall irradiation (mean MWHD for right-sided breast + IMC, chest wall + IMC, and IMC only were 14.5, 10.7, 9.1 Gy, respectively; mean MWHD for left-sided breast + IMC, chest wall + IMC, and IMC only were 18.6, 16.3, 14.2 Gy, respectively) (Table E2; available online at https://doi.org/10.1016/j.ijrobp.2018.10.025). MI rate increased with increasing MWHD (*P* trend, .011), and there was a 3.4-fold (95% CI, 1.54-7.62) increased rate for patients who received a MWHD ≥20 Gy compared with patients who received no radiation therapy (Table 2). Neither the use of chemotherapy nor the presence of cardiovascular risk factors significantly confounded the association between MWHD and MI rate (Table E3; available online at https://doi.org/10.1016/j.ijrobp.2018.10.025). When we restricted the analysis to irradiated cases and controls, similar rate increases for the different radiation dose categories were found (Table E4; available online at https://doi.org/10.1016/j.ijrobp.2018.10.025). Approximate 20-year cumulative risks were 0.9% (95% CI, 0.5-2.0), 1.8% (95% CI, 0.9-2.6), 2.1% (95% CI, 1.6-2.6), 2.5% (95% CI, 1.9-3.2), and 4.2% (95% CI, 1.2-7.2) for no radiation therapy and MWHD categories <2 Gy, 2 to 9 Gy, 10 to 19 Gy, and ≥20 Gy, respectively (Fig. 1).

**Table 2:** Associations between treatment characteristics and rate of myocardial infarction

Total	Cases		Controls		Rate ratio∗	95% CI	*P*†
	183 (N)	100 (%)‡	182 (N)	100 (%)‡			
Treatment model							
Surgery							
Breast conserving surgery (BCS)	82	44.8	87	47.8	1.00§	-	
Mastectomy	101	55.2	95	52.2	1.04	0.58-1.86	.90
Endocrine therapy							
No	167	91.3	166	91.2	1.00§	-	
Yes	16	8.7	16	8.8	0.71	0.29-1.75	.46
Chemotherapy (CT)							
No	140	76.5	132	72.5	1.00§	0.89-1.12	
CT, no anthracyclines	29	15.9	36	19.8	0.59	0.33-1.07	
Anthracycline-based CT	14	7.7	14	7.7	1.04	0.41-2.63	.23
Radiation therapy (RT)							
No	16	8.7	27	14.8	1.00‡	0.50-1.99	
RT, no IMC	51	27.9	65	35.7	1.20	0.67-2.18	
RT, IMC	116	63.4	90	49.5	2.45	1.97-3.05	.006
Mean whole heart dose (MWHD)‖							
Median MWHD (IQR) in Gy	8.9	4.8-15.0	8.5	4.3-12.2			
No RT	16	8.8	27	15.0	1.00§	0.52-1.91	
<2 Gy (mean 1 Gy)	29	15.9	34	18.9	1.44	0.84-2.48	
2-9 Gy (mean 7 Gy)	62	34.1	62	34.4	1.72	1.23-2.42	
10-19 Gy (mean 14 Gy)	57	31.3	48	26.7	2.06	1.40-3.02	
≥20 Gy (mean 26 Gy)	18	9.9	9	5.0	3.42	1.54-7.62	.011¶

*Abbreviations:* CI = confidence interval; IMC = internal mammary chain; IQR = interquartile range; MI = myocardial infarction.

∗ Univariable rate ratios for MI for different levels of each factor were calculated using logistic regression conditioning on strata defined by the matching variables.

† *P* value for difference between treatment characteristics of cases and controls, calculated within strata (defined by matching variables).

‡ Percentages may not total 100 because of rounding.

§ Reference category.

‖ This model includes 362 patients. Three patients were dropped; for one irradiated case and one irradiated control, dosimetry was not performed. One control was dropped because it was the only patient left in the stratum.

¶ *P* for trend across categories.

### Cardiovascular risk factors

Hypertension and a BMI ≥30, recorded at BC diagnosis, were the only individual patient-related cardiovascular risk factors significantly associated with an increased MI rate (Table E5; available online at https://doi.org/10.1016/j.ijrobp.2018.10.025). However, the presence of at least 1 cardiovascular risk factor (angina pectoris, chronic obstructive pulmonary disease, diabetes, hypertension, smoking, BMI ≥30) at BC diagnosis was associated with an RR of 1.70 (95% CI, 1.13-2.55) compared with no cardiovascular risk factors. When cardiovascular risk factors ever diagnosed (ie, during follow-up as well as at BC diagnosis) were taken into account, MI rate was higher (RR, 1.86; 95% CI, 1.19-2.90) (Table E5; available online at https://doi.org/10.1016/j.ijrobp.2018.10.025).

### Radiation dose-response relationship

**Fig. 2:**
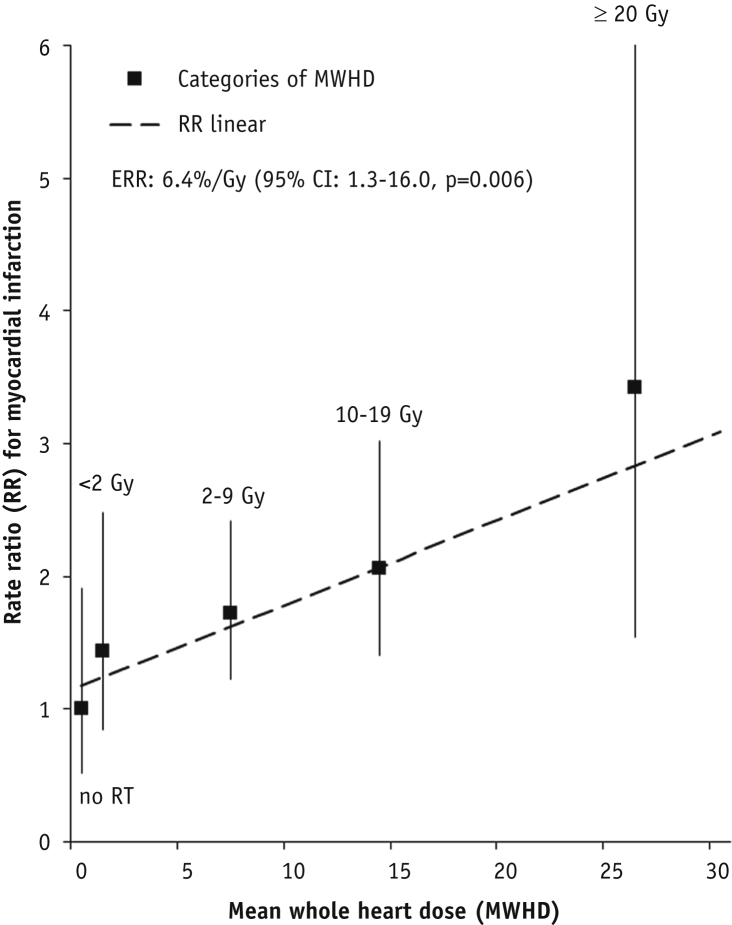
Dose-response relationship between rate of myocardial infarction and mean whole heart dose. The regression line is the best fitting linear dose response relationship (RR = κ[1.18 + 0.06389*MWHD]; *P* = .006). This results in an ERR of 6.4%/Gy (95% CI, 1.3-16.0). Squares indicate point estimates for dose categories (no RT, <2 Gy, 2-9 Gy, 10-19 Gy, ≥20 Gy; Table 2) and are plotted at the mean MWHD of each dose category. No significant departure from linearity was identified. The sum of the squared distances between the point estimates for dose categories was used to find the best fit of the linear dose-response to the categorical estimates, resulting in a linear RR of 1.18 at 0 Gy. *Abbreviations:* CI = confidence interval; ERR = excess rate ratio; MWHD = mean whole heart dose; RR = rate ratio; RT = radiation therapy.

A linear dose-response relationship between MWHD and MI rate was found. The ERR for MI increased by 6.4%/Gy (95% CI, 1.3%-16.0%; Fig. 2). A model that allowed for curvature gave no significant improvement in fit. When considering potential modifiers of the effect of MWHD on MI rate (Table 3), we found that ERRs were highest in women aged <45 years at BC diagnosis (24.2%/Gy; 95% CI, 4.4%-82.3%) and tended to decrease with older age (ERR_≥50-70years_, 2.5%/Gy; 95% CI, −1.4% to 11.9%) (*P*_interaction_ based on trend test, .07; *P*_interaction_ based on test for homogeneity between ERR_<45years_ and ERR_≥50-70years_, .054). ERRs increased with longer follow-up (*P*_interaction_ based on trend test, .25; *P*_interaction_ based on test for homogeneity between ERR_<10years_ and ERR_≥15years_, 0.053). The dose-response relationship for women with and without cardiovascular risk factors at BC diagnosis was similar (*P*_interaction_ > .50). Also, when considering cardiovascular risk factors ever diagnosed, results did not change.

**Table 3:** Excess rate ratios of myocardial infarction per Gy mean whole heart dose for subgroups

	Cases (N)	Controls (N)	ERR (%)	95% CI	*P*
All patients	183	182	6.4	1.3-16.0	0.006∗
Age at BC diagnosis					
<45 y	40	41	24.2	4.4-82.3	
45-49 y	47	47	11.1	1.2-40.1	
50-70 y	96	94	2.5	−1.4 to 11.9	.07†
Year of BC diagnosis					
1970-1979	41	41	4.0	−3.0 to 30.2	
1980-1989	95	92	8.0	0.8-26.2	
1990-1999	29	34	10.5	0.0-40.8	
≥2000	18	15	-1.6	−5.1 to 21.9	>.50†
Time to MI/cutoff date					
<10 y	44	44	-0.1	−2.9 to 9.5	
10-14 y	60	59	7.2	−0.8 to 32.3	
≥15 y	79	79	15.1	2.9-49.3	.25†
Chemotherapy (CT)					
No	140	132	9.9	2.4-25.5	
CT, no anthracyclines	29	36	1.5	−2.4 to 24.6	
Anthracycline-based CT	14	14	9.0	−4.2 to 113	>.50‡
Cardiovascular risk factors at BC diagnosis					
No	77	101	5.1	−0.3 to 17.3	
Yes	106	81	7.6	0.1-27.6	>.50‡
Cardiovascular risk factors ever diagnosed					
No	51	75	3.9	−1.6 to 18.7	
Yes	132	107	7.3	0.9-22.0	>.50‡
Smoking					
Never smoked	96	106	6.8	0.4-21.0	
Ever smoked	87	76	5.4	−0.7 to 21.5	>.50‡

*Abbreviations:* BC = breast cancer; CI = confidence interval; ERR = excess rate ratio; MI = myocardial infarction.

∗ *P* for test of ERR = 0.

† *P* for interaction based on trend test with time/age as a continuous variable.

‡ *P* for interaction based on test for homogeneity across categories.

### Whole heart dose-volume parameters

MWHD, mean left ventricle dose, and whole heart dose-volume measures were highly correlated (Pearson correlation coefficients ranged from 0.69 to 0.96). No other confounders of the relationship between V5 to V40 and MI rate were identified. In univariable models, MI rates were significantly increased when ≥30% of the heart received 5 to 30 Gy (RRs, 1.97-2.86; Table 4). The addition of MWHD as a continuous parameter to the separate dose-volume models did not significantly improve the fit of the models. Furthermore none of the dose-volume parameters was a better predictor than MWHD for the development of MI (ie, the deviance of ERR models was similar or worse when the dose-volume parameters were used to predict MI instead of the MWHD). Also, the addition of V5 to V40 to the linear odds model for MWHD did not significantly improve the fit of the ERR model (data not shown). No evidence for effect modification by any of the dose-volume parameters V5 to V40 was found (data not shown).

**Table 4:** Associations between percentage of heart volume receiving ≥5-40 Gy and rate of myocardial infarction

Percentage of heart volume receiving ≥5-40 Gy (V_5_-V_40_)	Median value (IQR)	Cases		Controls		Rate ratio∗	95% CI†	*P*‡
Total§		170 (N)	100 (%)‖	169 (N)	100 (%)‖			
V_5_	40.2 (11.1-87.6)							
<10%		44	25.9	62	36.7	1.00¶	0.68-1.47	-
10%-29%		29	17.1	35	20.7	1.20	0.73-1.99	.57
≥30%		97	57.1	72	42.6	2.02	1.43-2.85	.008
V_10_	37.1 (8.8-60.7)							
<10%		63	37.1	88	52.1	1.00¶	0.70-1.43	-
10%-29%		21	12.4	15	8.9	1.96	0.99-3.87	.09
≥30%		86	50.6	66	39.1	1.97	1.40-2.78	.007
V_15_	29.3 (8.0-40.5)							
<10%		64	37.7	91	53.8	1.00¶	0.72-1.39	-
10%-29%		22	12.9	15	8.9	2.01	1.03-3.93	.07
≥30%		84	49.4	63	37.3	2.05	1.41-2.98	.005
V_20_	17.1 (7.4-36.5)							
<10%		73	42.9	97	57.4	1.00¶	0.71-1.41	-
10%-29%		34	20.0	33	19.5	1.42	0.87-2.30	.25
≥30%		63	37.1	39	23.1	2.28	1.53-3.41	.002
V_25_	10.7 (6.8-19.1)							
<10%		77	45.3	104	61.5	1.00¶	0.73-1.37	-
10%-29%		53	31.2	46	27.2	1.60	1.04-2.45	.08
≥30%		40	23.5	19	11.2	2.86	1.67-4.90	.001
V_30_	6.6 (4.4-11.4)							
<10%		119	70.0	140	82.8	1.00¶	0.78-1.29	-
10%-29%		25	14.7	14	8.3	2.15	1.11-4.13	.033
≥30%		26	15.3	15	8.9	2.12	1.12-4.01	.033
V_35_	3.0 (1.2-6.2)							
<10%		132	77.7	149	88.2	1.00¶	0.76-1.31	-
10%-29%		24	14.1	12	7.1	2.32	1.17-4.58	.025
≥30%		14	8.2	8	4.7	2.11	0.89-5.03	.11
V_40_	0.3 (0-5.4)							
<10%		143	84.1	154	91.1	1.00¶	0.88-1.13	-
10%-29%		23	13.5	14	8.3	1.77	0.88-3.57	.12
≥30%		4	2.4	1	0.6	5.29	0.57-49.4	.15

*Abbreviations:* CI = confidence interval; IQR = interquartile range; MI = myocardial infarction.

∗ Univariable rate ratios for MI for different levels of each factor were calculated using logistic regression conditioning on strata defined by the matching variables.

† Wald method CIs were used to derive CIs for each category, including the reference category.

‡ *P* value for difference of dose-volume categories of cases and controls, calculated within strata (defined by matching variables), with <10% as a fixed reference group.

§ This model includes 339 patients. Twenty-six patients were dropped: for one irradiated case and one irradiated control, dosimetry was not performed. For 22 patients (11 cases and 11 controls) it was not possible to estimate dose-volume parameters study because they had a combination of orthovoltage and electron/megavoltage treatment (manual planning). One case and one control were additionally dropped because they were the only patient left in the stratum.

‖ Percentages may not total 100 because of rounding.

¶ Reference category.

## Discussion

In this study we found that MI rate increases linearly by 6.4% per Gy increase in MWHD in BC survivors with a median age of 50 years at BC diagnosis and that there was a 3.4-fold increased MI rate at a MWHD of ≥20 Gy. We did not find any evidence that the percentage increase in MWHD rate per Gy differed by presence or absence of cardiovascular risk factors at BC diagnosis. These findings are consistent with the previously published radiation dose-response relationship between MWHD and MI rate in BC survivors, which found an MI rate of 7.4% per Gy increase in MWHD for women both with and without cardiovascular risk factors at time of radiotherapy.^14^ Moreover, similar results were reported in a study assessing the dose-response relationship for MWHD and the risk of IHD in Hodgkin lymphoma (HL) survivors.^15^

Our results suggest that ERR per Gy was higher for the youngest women (aged <45 years at BC diagnosis) and this effect had borderline significance (*P*_interaction_ = .054). It is notable that Van Nimwegen et al^15^ also found ERRs to be highest in patients treated for HL at the youngest ages. However, ERRs for HL patients treated at an age comparable to our youngest age group were lower than we identified (ie, 4.2%/Gy in HL patients irradiated between ages 36 and 50 vs 24.2%/Gy in our patients irradiated at <45 years, median 37 years).^15^ In a case-control study by Darby et al,^14^ no significant effect of age was found on the slope of the dose-response relationship between MWHD and major coronary event rate. Some cohort studies in HL survivors and BC survivors have, however, reported that younger age is associated with higher relative risks of radiation-induced heart disease.^24^ Whether radiation therapy is associated with larger percentage increases in the MI rate per Gy MWHD in very young BC patients (ie, treated before age 40 years) remains an important gap in knowledge and requires further investigation.

In some earlier studies it was found that radiation therapy was not associated with IHD during the first 10 years after exposure,^16^, ^17^ but evidence is now emerging that radiation therapy can lead to increased IHD rates within the first 5 years.^14^, ^25^ We, however, did not identify increased MI rates within the first 10 years after BC diagnosis (ERR_<10years_, −0.1%; 95% CI, −2.9% to 9.5%). This could be due to the inclusion of relatively young women in our study; almost 90% of our patients were aged <60 years at BC diagnosis. In contrast, in the study by Darby et al^14^ almost half of included women were aged ≥60 years at BC diagnosis. In another study, presenting increased rates of major acute coronary events in relation to MHD within 9 years after radiation therapy, median age of included women was also higher (59 years; range, 26-84 years) compared with the median age of our study population.^25^ Strong conclusions about the presence of latency in the development of radiation-induced IHD, however, cannot be drawn from our data because follow-up time was not a significant effect modifier and the number of included cases is rather small to assess the effects in different follow-up groups.

We found that none of the whole heart dose-volume parameters was a better predictor than MWHD for the development of MI and we found no evidence for effect modification of the dose-response relationship by any of the dose-volume measures. In a recent study among women treated during 2005 to 2008 for BC or ductal carcinoma in situ, the left ventricle V5 (LV-V5) seemed to be a better predictor of acute coronary events than MWHD.^25^ An important limitation of that study is, however, that there were only 30 patients who developed an event, whereas we included 183 cases of MI after BC radiation therapy. The women included in the 2 studies were also irradiated in different eras, 1970s to 2000s in our study and 2005 to 2008 in the other study, so radiation techniques and the distribution of MWHD are different. Hence we cannot compare our findings directly.

More information is needed on whether, for a given MWHD, the dose-response relationship differs in situations in which the heart is partly irradiated (“a lot to a little”) versus situations in which the heart is homogeneously irradiated (“a little to a lot”).^26^ Our data, however, do not allow us to study whether, for a given MWHD, dose inhomogeneity across the heart is a significant predictor of MI. Variation in the dose-volume measures was limited given that most women received a high dose to a small part of the heart (“a lot to a little”) and MWHD and our dose-volume measures were strongly correlated. Furthermore the number of cases in each dose-volume category was too low to draw conclusions around the effects of exposing varying proportions of the heart to a certain radiation dose. Schneider et al^26^ investigated this topic by modeling the dose-response relationship using relative seriality models to extrapolate to situations in which no data are currently available. They suggested that the dose-response relationship may be sigmoidal rather than linear when the heart is homogeneously irradiated (“a little to a lot”). In a recent paper by Hahn et al,^27^ among HL survivors it was also found that increasing dose inhomogeneity was a significant predictor of cardiac toxicity and ischemic cardiac events. Studies including women irradiated with CT-based radiation therapy planning and women who received intensity modulated radiation therapy will further enable investigation of the role of dose-volume parameters in relation to the development of IHD among BC survivors.

A limitation of our study was that we were unable to use dosimetry data derived from CT-planning scans of the patients themselves because most women were treated before the era of CT planning. Instead, individual radiation therapy regimens for irradiated patients were reconstructed on the CT scan of a typical patient and thus the cardiac doses may differ from presented estimates, principally because of variation in anatomy (Methods E1; available online at 10.1016/j.ijrobp.2018.10.025). The magnitude of these differences will be similar for cases and controls, but it will differ between different regimens. The nature of this error, known as a Berkson error, has been found to cause no bias in the resulting dose-response relationship.^28^ Furthermore, it should be noted that cumulative MI risks by dose categories were estimated based on the cumulative MI risk identified in our BC cohort. Over the past decade, decreases in MI rate have been reported in the general population. Because the cumulative absolute risk is affected by the background MI rate, the cumulative incidence for future BC patients is likely to be lower than presented here.

An important strength is that this large case-control study is nested within a well-characterized cohort for which comprehensive oncologic and cardiovascular follow-up information was available. We ascertained MI diagnosis through the patients’ GP, who in the Netherlands routinely receives medical correspondence from attending physicians or the treating cardiologist. Furthermore, individual patient dosimetry was performed and we found no evidence for selection of irradiated patients according to health status, because our sensitivity analyses restricted to irradiated patients indicated similar risk estimates for categories of MWHD.

## Conclusions

We found a linear dose-response relationship between MWHD and MI rate in a population of younger BC patients (median age of 50 years at BC diagnosis), suggesting that there is no apparent threshold dose below which MI rate is not increased. Our findings stress the importance of maximally reducing MWHD using modern radiation techniques to optimize the cardiovascular health of BC survivors.
