# Supplementary material for: Radiation Dose-Response for Risk of Myocardial Infarction in Breast Cancer Survivors
Source: Int J Radiat Oncol Biol Phys. 2019 Mar 1;103(3):595–604. doi: 10.1016/j.ijrobp.2018.10.025 (PMC6361769; doi:10.1016/j.ijrobp.2018.10.025)
Supplement: Supplementary Material [file mmc1.docx]

**Supplementary methods I**

Female breast cancer (BC) patients (stage I-IIIA or ductal carcinoma in situ [DCIS]) diagnosed from 1970-2009 were selected from the hospital-based registries of the … All patients had to have received surgery. Data collection from the registries and medical files included the following variables: date of birth, date of BC diagnosis, tumor histology, stage, type of surgery, radiation fields, chemotherapy regimen, hormonal treatment, date of first recurrence and distant metastasis, date, diagnosis and treatment of previous and subsequent malignancies, history of cardiovascular disease (CVD) before BC diagnosis, dates and diagnoses of cardiovascular events, smoking, hypertension, diabetes mellitus, hypercholesterolemia, date of last known medical status, and cause of death (according to the International Classification for Diseases, 10th revision). Because data collection on CVD incidence through questionnaires to general practitioners and cardiologists is very labour intensive, and because we were interested in long-term cardiovascular disease risks following radiotherapy and chemotherapy, we collected information only for selected patients. Selection of patients was always random within each age stratum and independent of CVD diagnosis, as the hospital-based registries did not contain any data on CVD. For patients diagnosed between 1970 and 1986, CVD information was acquired only for selected ten-year survivors [1]. For patients diagnosed between 1987 and 2009, we collected CVD information for selected one-year survivors [2]. For the years of diagnosis 2001 to 2009 all one-year survivors were included. To complete cardiovascular follow-up in the entire cohort, letters were sent to general practitioners and cardiologists. In routine clinical practice, medical correspondence from attending physicians is sent to the general practitioner. Records are preserved by the general practitioner throughout a patient’s life and for at least 15 years after a patient’s death. For patients treated before 2000, complete follow-up information to at least January 1, 2009 was available for 82% of the study cohort. For patients treated in 2000-2009, complete follow-up information through at least January 1, 2012, was available for 71% of the study cohort. Median follow-up duration was 14 years for the entire cohort; 23 years for patients diagnosed with breast cancer ≤1986 and 12 years for patients diagnosed >1986.

[1] …

[2] …

**Supplementary methods II**

For the women in this study CT-planning was only available from 2005 onwards. Individual anatomical information was not available. Cardiac doses were estimated retrospectively by two of the authors (….).

*Chart categorisation*

Information was abstracted from 314 radiotherapy charts including: surgery type, target definition, field borders, total dose and dose per fraction, beam energy and the use of shielding, wedges and bolus. In total, 52 regimens were received, 28 regimens for left-sided and 24 for right-sided BC (Supplemental table 1). The main regimen categories were: tangential (35%, n=109), megavoltage internal mammary chain (IMC) (25%, n=80), orthovoltage or mixed orthovoltage/megavoltage IMC (28%, n=89), and electron chest wall or IMC (12%, n=36). Wide tangential or partially wide tangential techniques were not used in this study. Patients were identified as having IMC irradiation if dedicated anterior parasternal beams were used.

*Selection of a 'typical CT-scan'*

Ten CT-planning scans were randomly selected from women referred for BC radiotherapy in 2010. The treatment position for all women was supine, with both arms above the head. Slice thickness for each scan was 3mm, and intravenous contrast was not used. The whole heart and left ventricle were contoured on each of the 10 scans [1]. To simulate mastectomy duplicate copies of the CT were created and the relevant breast was virtually removed from the dose calculations. To select a ‘typical CT-scan’, first the most commonly used left-sided regimen was identified from the charts. This was a midline tangential regimen consisting of two opposing symmetrical fields with alignment of the posterior field borders which was used from the 1980s up to the early 2000s. Second, the regimen was reconstructed on each of the ten CT-scans and whole heart doses were reviewed. Third, anatomical features which may influence cardiac doses from BC radiotherapy were measured, including: sternal length, heart volume, chest wall separation distance and the Haller index (ratio of height between the anterior spine and posterior sternum to the transverse width of the heart). The 'typical CT-scan' was the scan with a MWHD closest to average (MWHD ‘typical CT-scan’: 4.8 Gy, average MWHD ten CT-scans: 4.8 Gy (range 1.9 – 9.1 Gy)). It was not atypical for any of the anatomical factors reviewed.

*Regimen reconstruction*

All other regimens identified from the radiotherapy charts were then reconstructed on the ‘typical CT-scan’. Dose distributions were generated for cobalt, electron and megavoltage beams using modern 3-dimensional CT treatment planning (Varian EclipseTM Treatment Planning System [TPS] version 10.0.39 [Varian Medical Systems, Palo Alto, USA]). The analytical anisotropic algorithm was used to calculate cardiac doses for photon plans, Monte Carlo was used for electron plans, and pencil beam for cobalt plans. Dose distributions from orthovoltage fields were generated using manual planning. MWHD, MLVD, V_5_ to V_40_ were estimated using dose volume histograms.

*Allocation of doses to individual women*

Doses were estimated for each individual woman using the total dose (100%) she received as recorded in individual radiotherapy charts and the dose volume histogram of the regimen she received.

*Limitations*

The estimated cardiac doses are subject to several sources of uncertainty. Many of these uncertainties are common to all radiotherapy CT-planning studies and include delineation error, dose calculation algorithm error, set-up error, and cardiac and respiratory motion during treatment. In this study of women for whom no anatomical information is available another important source of uncertainty is inter-patient differences in anatomy.  For left tangential radiotherapy inter-patient dose variability was assessed by reconstructing midline tangents on ten CT scans. The difference between the highest and lowest mean dose estimated for the whole heart and LV was 7.2 Gy and 10.7 Gy respectively.  For right tangential radiotherapy inter-patient dose variability was assessed by reconstructing fields on five of the ten CT-scans. The inter-patient dose variability for the whole heart and LV was 0.5 Gy and 0.2 Gy respectively.

[1] ….

| **Supplemental table 1. Radiotherapy techniques identified from 314 radiotherapy charts of women treated for breast cancer in …. during 1970-2009** | | | | | |
| --- | --- | --- | --- | --- | --- |
|  |  |  |  | *Number of women* | |
| *Years of radiotherapy* | *Field arrangement* | *Beam energies* | *Prescribed dose (100%) Gy^*^* | *Left^†^* | *Right^†^* |
| ***Tangential (109 charts)*** | | | | | |
| 1980s | Tangents | Co^60^ | 50.0 | 3 | 3 |
| 1980s | Tangents, parasternal boost | 6 MV, 10 MeV | 45.0/9.0 | 2 | 0 |
| 1980s-2000s | Tangents | 6 MV | 50.0 | 42 | 36 |
| 2000s | Tangents | 6 MV | 50.0 | 11 | 12 |
| ***Megavoltage IMC (80 charts)*** | | | | | |
| *Megavoltage* | | | | | |
| 1970s-1980s | Direct IMC | Co^60^ | 43.0 | 3 | 2 |
| 1970s-1980s | Direct IMC, matching direct chest wall | Co^60^/9 MeV | 40.0/37.6 | 6 | 5 |
| 1970s-1980s | Direct IMC, matching tangents | Co^60^/6 MV | 53.8/50.0 | 11 | 17 |
| 1970s-1990s | Direct IMC, direct SCF/axilla/lateral thorax | 6 MV/8 MV | 40.5/44.5 | 4 | 3 |
| 1980s | Direct IMC, matching direct chest wall | Co^60^/6 MV/9 MeV | 35.4/14.1/40.0 | 0 | 1 |
| 1990s | Direct IMC, matching direct chest wall | 6 MV/9MeV | 46.1/40.0 | 0 | 1 |
| 1980s-2000s | Direct IMC, matching tangents | 6 MV/6 MV | 50.0/50.0 | 1 | 4 |
| *Mixed megavoltage/electron* | | | | | |
| 1980s | Direct IMC, matching tangents | Co^60^/12 MeV/6 MV | 28.0/24.0/50.0 | 2 | 0 |
| 1980s | Direct IMC | Co^60^/12 MeV | 25.5/26.0 | 1 | 1 |
| 1980s | Direct IMC, matching direct chest wall | Co^60^/12 MeV/9 MeV | 20.0/18.8/37.6 | 1 | 0 |
| 1980s-1990s | Direct IMC, matching tangents | 6 MV/12 MeV/6 MV | 27.2/24.0/50.0 | 1 | 4 |
| 1990s | Direct IMC, matching direct chest wall | 6 MV/12 MeV/9 MeV | 22.5/22.5/40.0 | 3 | 1 |
| 1990s-2000s | Direct IMC | 6 MV/12 MeV | 18.7/21.4 | 1 | 3 |
| 2000s | Direct IMC, matching tangents | 6 MV/12 MeV/6 MV | 18.0/32.0/50.0 | 2 | 1 |
| 2000s | Oblique IMC, matching tangents | 6 MV/12 MeV/6 MV | 18.0/32.0/50.0 | 1 | 0 |
| ***Orthovoltage or mixed orthovoltage/megavoltage IMC (89 charts)*** | | | | | |
| 1970s-1980s | Direct IMC | 250 keV | 37.2 | 29 | 27 |
| 1970s-1990s | Direct IMC, matching tangents | 250 keV/Co^60^ | 37.2/30.0 | 1 | 0 |
| 1970s | Direct IMC, matching direct chest wall | 250 keV/9 MeV | 37.2/40.0 | 3 | 7 |
| 1980s | Direct IMC | 250 keV/Co^60^ | 17.2/22.7 | 10 | 6 |
| 1990s | Direct IMC | 250 keV/6 MV | 16.8/21.9 | 3 | 0 |
| 1990s | Direct IMC, matching tangents | 250 keV/6 MV/6 MV | 23.5/24.0/50.0 | 1 | 2 |
| ***Electron chest wall or IMC (36 charts)*** | | | | | |
| 1970s-1980s | Direct IMC | 12 MeV | 45.0 | 9 | 7 |
| 1970s-1980s | Direct IMC, matching tangents | 12 MeV/6 MV | 45.0/45.0 | 8 | 3 |
| 1970s-1980s | Direct IMC, matching direct chest wall | 12 MeV/9 MeV | 45.0/45.0 | 2 | 1 |
| 1970s-1990s | Direct chest wall | 9 MeV | 45.0 | 2 | 1 |
| 1970s-2000s | Direct chest wall, direct SCF/axilla/lateral thorax | 9 MeV/8 MV | 45.0/53.0 | 1 | 2 |
| Abbreviations: IMC: internal mammary chain keV: kilovoltage, MV: megavoltage; MeV: mega electron-volts, SCF: supraclavicular fossa, contra: contralateral, ipsi: ipsilateral, Co60: cobalt 60  Highlighted regimens did not include the IMC but all other regimens did.  * Prescribed total dose (100%) to the target tissues. For direct regimens this was the Dmax. For tangents this was the dose delivered to the centre of the breast or chest wall.  † Number of women irradiated for left breast cancer and right breast cancer. | | | | | |

**
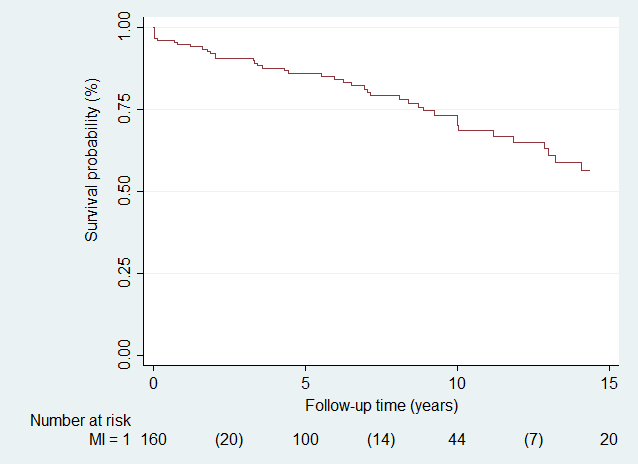
**

**Supplemental figure 1: Survival after myocardial infarction diagnosis. Calculated using the Kaplan-Meier method.**

| **Supplemental table 2: Mean and median mean whole heart dose, V5 and V25 by prescribed radiation field for all irradiated women (cases and controls)** | | | | | | | |
| --- | --- | --- | --- | --- | --- | --- | --- |
|  | N | Mean MWHD ± SD | Median MWHD (IQR) | N | Median V5 (IQR) | Median V25 (IQR) | |
| Total | 314^*^ | 9.5 ± 7.4 | 8.9 (4.3-13.3) | 292^†^ | 39.6 (11.1-87.6) | 10.5 (6.8-19.1) | |
|  | Right-sided BC | | | | | | |
| Breast | 51 | 0.6 ± 0.2 | 0.6 (0.6-0.7) | 51 | 0 (-) | 0 (-) | |
| Breast + IMC | 30 | 14.5 ± 3.8 | 16.1 (13.2-16.6) | 29 | 47.5 (42.0-48.5) | 35.3 (31.8-35.8) | |
| Chest wall | 3 | 2.4 ± 0.6 | 2.8 (1.7-2.8) | 3 | 11.2 (9.2-11.3) | 1.9 (1.1-2.0) | |
| Chest wall + IMC | 17 | 10.7 ± 3.5 | 10.5 (8.9-13.0) | 16 | 46.4 (44.9-90.1) | 15.1 (10.7-31.2) | |
| IMC only | 49 | 9.1 ± 2.3 | 8.9 (8.7-9.4) | 43 | 90.0 (40.3-90.1) | 10.6 (9.9-11.0) | |
|  | Left-sided BC | | | | | | |
| Breast | 56 | 4.0 ± 1.3 | 4.8 (4.3-4.8) | 56 | 11.8 (11.1-11.8) | 7.1 (6.9-7.1) | |
| Breast + IMC | 30 | 18.6 ± 9.6 | 17.8 (8.3-28.8) | 29 | 81.4 (36.7-87.6) | 25.4 (11.8-66.7) | |
| Chest wall | 3 | 4.6 ± 1.5 | 4.0 (3.5-6.3) | 3 | 19.3 (18.4-24.8) | 5.5 (4.3-6.8) | |
| Chest wall + IMC | 15 | 16.3 ± 6.9 | 16.1 (12.2-22.5) | 15 | 85.2 (65.9-86.7) | 20.1 (18.4-51.4) | |
| IMC only | 60 | 14.2 ± 5.1 | 12.2 (12.1-16.5) | 47 | 98.2 (80.8-99.0) | 18.4 (16.2-19.1) | |
| Abbreviations: MWHD, mean whole heart dose, SD, standard deviation, IQR, interquartile range, IMC, internal mammary chain.  * In total, for 314/316 irradiated, *unique* individuals mean whole heart dose was estimated.  † In total, for 292/316 irradiated, *unique* individuals dose-volume parameters were estimated. For 22 patients it was not possible to estimate dose-volume parameters study as they had a combination of orthovoltage and electron/megavoltage treatment (manual planning). | | | | | | |  |

| **Supplemental table 3 : Associations between mean whole heart dose and rate of myocardial infarction, including adjusted rate ratios** | | | | | | | | | | | | |
| --- | --- | --- | --- | --- | --- | --- | --- | --- | --- | --- | --- | --- |
|  | **Cases** |  | **Controls** |  | **Rate ratio^*^** | **95% CI** | **Adjusted rate ratio^¥^** | **95% CI** | **Adjusted rate ratio^II^** | **95% CI** | **Adjusted rate ratio^**^** | **95% CI** |
| Total | 182^§^ (N) | 100 (%) | 180^¶^ (N) | 100 (%) |  |  |  |  |  |  |  |  |
| Median MWHD (IQR) in Gy | 8.9 | 4.8-15.0 | 8.5 | 4.3-12.2 |  |  |  |  |  |  |  |  |
| No RT | 16 | 8.8 | 27 | 15.0 | 1.00^‡^ | 0.52-1.91 | 1.00^‡^ | 0.52-1.92 | 1.00^‡^ | 0.52-1.92 | 1.00^‡^ | 0.52-1.92 |
| <2 Gy (mean 1 Gy) | 29 | 15.9 | 34 | 18.9 | 1.44 | 0.84-2.48 | 1.39 | 0.80-2.42 | 1.46 | 0.84-2.53 | 1.41 | 0.81-2.48 |
| 2-9 Gy (mean 7 Gy) | 62 | 34.1 | 62 | 34.4 | 1.72 | 1.23-2.42 | 1.77 | 1.26-2.48 | 1.65 | 1.17-2.32 | 1.69 | 1.20-2.38 |
| 10-19 Gy (mean 14 Gy) | 57 | 31.3 | 48 | 26.7 | 2.06 | 1.40-3.02 | 2.15 | 1.46-3.18 | 2.00 | 1.35-2.93 | 2.07 | 1.40-3.07 |
| ≥20 Gy (mean 26 Gy) | 18 | 9.9 | 9 | 5.0 | 3.42 | 1.54-7.62 | 3.63 | 1.63-8.12 | 3.41 | 1.52-7.62 | 3.58 | 1.60-8.03 |
| P-value^†^ |  |  |  |  | 0.011 |  | 0.006 |  | 0.015 |  | 0.009 |  |
| ERR/Gy |  |  |  |  | 6.4 | 1.3-16.0 | 7.5 | 1.8-18.4 | 6.1 | 1.1-15.4 | 7.0 | 1.5-17.5 |
| Abbreviations: CI, confidence interval, MWHD, mean whole heart dose, IQR, inter quartile range, RT, radiotherapy.  § For one irradiated case dosimetry was not performed.  ¶ For one irradiated control dosimetry was not performed. One control was additionally dropped, because it was the only patient left in the stratum.  * Rate ratios for MI for different levels of each factor were calculated using logistic regression conditioning on strata defined by the matching variables.  ‡ Reference category.  ¥ Adjusted for chemotherapy yes/no, in addition to stratification.  II Adjusted for cardiovascular risk factors (combined variable) at BC diagnosis, in addition to stratification.  ** Adjusted for cardiovascular risk factors (combined variable) at BC diagnosis and for chemotherapy yes/no, in addition to stratification.  † P-value for trend across categories. | | | | | | | | | | | | |

| **Supplemental table 4: Associations between mean whole heart dose and rate of myocardial infarction risk in irradiated cases and irradiated controls** | | | | | | | |
| --- | --- | --- | --- | --- | --- | --- | --- |
|  | **Cases** |  | **Controls** |  | **Rate ratio*** | **95% CI** | **P-value** |
| Total | 165 (N) | 100 (%) | 163 (N)^¶^ | 100 (%) |  |  |  |
| Median MWHD dose (IQR) in Gy | 8.9 | (4.8-15.0) | 8.3 | (4.0-12.2) |  |  |  |
| <2 Gy (mean 1 Gy) | 29 | 17.6 | 37 | 22.7 | 1.00^‡^ | 0.59-1.70 |  |
| 2-9 Gy (mean 7 Gy) | 62 | 37.6 | 68 | 41.7 | 1.23 | 0.89-1.70 |  |
| 10-19 Gy (mean 14 Gy) | 56 | 33.9 | 50 | 30.7 | 1.52 | 1.03-2.25 |  |
| ≥20 Gy (mean 26 Gy) | 18 | 10.9 | 8 | 4.9 | 3.03 | 1.32-6.98 | 0.029**^†^** |
| ERR/Gy |  |  |  |  | 6.7 | 1.0-19.6 |  |
| Abbreviations: CI, confidence interval, MWHD, mean whole heart dose, IQR, interquartile range.  * Rate ratios for MI for different levels of each factor were calculated using logistic regression conditioning on strata defined by the matching variables.  ¶ Irradiated cases were matched to irradiated controls only and the number of controls per dose category may differ from the number of controls per dose category shown in table 2.  † P for trend across categories.  ‡ Reference category. | | | | | | | |

| **Supplemental table 5. Associations between patient-related risk factors and rate of myocardial infarction** | | | | | | | | |
| --- | --- | --- | --- | --- | --- | --- | --- | --- |
|  | **Cases** |  | **Controls** |  | **Rate ratio^¶^** | **95% CI** | **P-value^†^** |  |
| **Total** | 183 (N) | 100 (%)^*^ | 182 (N) | 100 (%)^*^ |  |  |  |  |
| History of hypertension^II^ |  |  |  |  |  |  |  |  |
| No^**^ | 159 | 86.9 | 175 | 96.2 | 1.00^‡^ | - |  |  |
| Yes | 24 | 13.1 | 7 | 3.8 | 3.85 | 1.60-9.25 | 0.003 |  |
| History of angina pectoris^II^ |  |  |  |  |  |  |  |  |
| No^**^ | 179 | 97.8 | 180 | 98.9 | 1.00^‡^ | - |  |  |
| Yes | 4 | 2.2 | 2 | 1.1 | 1.98 | 0.35-11.09 | 0.44 |  |
| History of diabetes^II^ |  |  |  |  |  |  |  |  |
| No^**^ | 177 | 96.7 | 181 | 99.5 | 1.00^‡^ | - |  |  |
| Yes | 6 | 3.3 | 1 | 0.5 | 6.25 | 0.74-52.72 | 0.09 |  |
| History of COPD^II^ |  |  |  |  |  |  |  |  |
| No^**^ | 182 | 99.5 | 180 | 98.9 | 1.00^‡^ | - |  |  |
| Yes | 1 | 0.5 | 2 | 1.1 | 0.50 | 0.05-5.51 | 0.57 |  |
| Body mass index (BMI)^II^ |  |  |  |  |  |  |  |  |
| BMI<25 | 77 | 42.1 | 94 | 51.6 | 1.00^‡^ | 0.74-1.36 |  |  |
| BMI 25-29 | 43 | 23.5 | 36 | 19.8 | 1.45 | 0.93-2.24 |  |  |
| BMI ≥30 | 14 | 7.7 | 6 | 3.3 | 2.90 | 1.11-7.56 |  |  |
| Unknown | 49 | 26.8 | 46 | 25.3 | 1.33 | 0.87-2.03 | 0.053 |  |
| Current smoking^II^ |  |  |  |  |  |  |  |  |
| No | 93 | 50.8 | 92 | 50.5 | 1.00^‡^ | 0.75-1.33 |  |  |
| Yes | 75 | 41.0 | 68 | 37.4 | 1.11 | 0.79-1.55 |  |  |
| Unknown | 15 | 8.2 | 22 | 12.1 | 0.68 | 0.35-1.31 | 0.67 |  |
| At least one cardiovascular risk factor^II^ |  |  |  |  |  |  |  |  |
| No | 77 | 42.1 | 101 | 55.5 | 1.00^‡^ | - |  |  |
| Yes | 106 | 57.9 | 81 | 44.5 | 1.70 | 1.13-2.55 | 0.011 |  |
| Ever smoking |  |  |  |  |  |  |  |  |
| No | 81 | 44.3 | 84 | 46.2 | 1.00^‡^ | 0.74-1.34 |  |  |
| Yes | 87 | 47.5 | 76 | 41.8 | 1.20 | 0.87-1.65 |  |  |
| Unknown | 15 | 8.2 | 22 | 12.1 | 0.71 | 0.37-1.37 | 0.43 |  |
| Cardiovascular risk factors ever diagnosed^¥^ |  |  |  |  |  |  |  |  |
| No | 51 | 27.9 | 75 | 41.2 | 1.00^‡^ | - |  |  |
| Yes | 132 | 72.1 | 107 | 58.8 | 1.86 | 1.19-2.90 | 0.007 |  |
| Abbreviations: CI, confidence interval, COPD, chronic obstructive pulmonary disease.  * Percentages may not total 100 because of rounding.  ¶ Rate ratios for MI for different levels of each factor were calculated using logistic regression conditioning on strata defined by the matching variables.  † P-value for difference between MI rate ratios of women with and without cardiac risk factors, calculated within strata (defined by matching variables) and excluding the unknown category.  ‡ Reference category.  II Cardiovascular risk factors recorded at BC diagnosis.  ** This category includes women with an unknown history of hypertension/angina/diabetes/COPD at BC diagnosis.  ¥ Combined variable including: angina pectoris, COPD, diabetes, hypertension, smoking, BMI≥30. | | | | | | | | |

| **Supplemental table 6: Associations between mean left ventricle dose and rate of myocardial infarction** | | | | | | |  |
| --- | --- | --- | --- | --- | --- | --- | --- |
|  | **Cases** |  | **Controls** |  | **Rate ratio^*^** | **95% CI** | **P-value^†^** |
| Total | 182^§^ (N) | 100 (%) | 180^¶^ (N) | 100 (%) |  |  |  |
| Median MLVD (IQR) in Gy | 2.2 | (1.7-8.9) | 2.2 | (1.3-8.7) |  |  |  |
| No RT | 16 | 8.8 | 27 | 15.0 | 1.00^‡^ | 0.52-1.92 |  |
| <2 Gy (mean 1 Gy) | 73 | 40.1 | 65 | 36.1 | 2.02 | 1.46-2.77 |  |
| 2-4 Gy (mean 3 Gy) | 28 | 15.4 | 19 | 10.6 | 2.92 | 1.56-5.47 |  |
| 5-9 Gy (mean 8 Gy) | 36 | 19.8 | 48 | 26.7 | 1.34 | 0.87-2.07 |  |
| ≥10 Gy (mean 17 Gy) | 29 | 15.9 | 21 | 11.7 | 2.53 | 1.45-4.43 | 0.45 |
| Abbreviations: CI, confidence interval, MLVD, mean left ventricle dose, IQR, interquartile range, RT, radiotherapy.  § For 1 case dosimetry was not performed.  ¶ Two controls were dropped; for one irradiated control, dosimetry was not performed. One control was dropped, because it was the only patient left in the stratum.  * Rate ratios for MI for different levels of each factor were calculated using logistic regression conditioning on strata defined by the matching variables.  † P for trend across categories.  ‡ Reference category. | | | | | | | |
